# Definitions and Measurements for Atypical Presentations at Risk for Diagnostic Errors in Internal Medicine: Protocol for a Scoping Review

**DOI:** 10.2196/56933

**Published:** 2024-03-25

**Authors:** Yukinori Harada, Ren Kawamura, Masashi Yokose, Taro Shimizu, Hardeep Singh

**Affiliations:** 1 Department of Diagnostic and Generalist Medicine Dokkyo Medical University Mibu Japan; 2 Center for Innovations in Quality, Effectiveness and Safety Michael E. DeBakey Veterans Affairs Medical Center Houston, TX United States; 3 Health Services Research Section, Department of Medicine Baylor College of Medicine Houston, TX United States

**Keywords:** atypical presentations, diagnostic errors, internal medicine, scoping review protocol, atypical presentation, high risk, data extraction, descriptive statistics, criteria, qualitative, content analysis, inductive approach, clinical informatics, clinical decision support

## Abstract

**Background:**

Atypical presentations have been increasingly recognized as a significant contributing factor to diagnostic errors in internal medicine. However, research to address associations between atypical presentations and diagnostic errors has not been evaluated due to the lack of widely applicable definitions and criteria for what is considered an atypical presentation.

**Objective:**

The aim of the study is to describe how atypical presentations are defined and measured in studies of diagnostic errors in internal medicine and use this new information to develop new criteria to identify atypical presentations at high risk for diagnostic errors.

**Methods:**

This study will follow an established framework for conducting scoping reviews. Inclusion criteria are developed according to the participants, concept, and context framework. This review will consider studies that fulfill all of the following criteria: include adult patients (participants); explore the association between atypical presentations and diagnostic errors using any definition, criteria, or measurement to identify atypical presentations and diagnostic errors (concept); and focus on internal medicine (context). Regarding the type of sources, this scoping review will consider quantitative, qualitative, and mixed methods study designs; systematic reviews; and opinion papers for inclusion. Case reports, case series, and conference abstracts will be excluded. The data will be extracted through MEDLINE, Web of Science, CINAHL, Embase, Cochrane Library, and Google Scholar searches. No limits will be applied to language, and papers indexed from database inception to December 31, 2023, will be included. Two independent reviewers (YH and RK) will conduct study selection and data extraction. The data extracted will include specific details about the patient characteristics (eg, age, sex, and disease), the definitions and measuring methods for atypical presentations and diagnostic errors, clinical settings (eg, department and outpatient or inpatient), type of evidence source, and the association between atypical presentations and diagnostic errors relevant to the review question. The extracted data will be presented in tabular format with descriptive statistics, allowing us to identify the key components or types of atypical presentations and develop new criteria to identify atypical presentations for future studies of diagnostic errors. Developing the new criteria will follow guidance for a basic qualitative content analysis with an inductive approach.

**Results:**

As of January 2024, a literature search through multiple databases is ongoing. We will complete this study by December 2024.

**Conclusions:**

This scoping review aims to provide rigorous evidence to develop new criteria to identify atypical presentations at high risk for diagnostic errors in internal medicine. Such criteria could facilitate the development of a comprehensive conceptual model to understand the associations between atypical presentations and diagnostic errors in internal medicine.

**Trial Registration:**

Open Science Framework; www.osf.io/27d5m

**International Registered Report Identifier (IRRID):**

DERR1-10.2196/56933

## Introduction

Diagnostic errors, defined as “the failure to (a) establish an accurate and timely explanation of the patient’s health problem(s) or (b) communicate that explanation to the patient” [1], is an important concern in improving patient safety and diagnostic excellence. According to a recent report, approximately 0.8 million people may become permanently disabled or die annually due to diagnostic errors in the United States [2]. While diagnostic errors can occur across care settings, internal medicine is one of the highest-risk care settings for diagnostic errors [3,4]. Internal medicine physicians, as well as surgery and emergency medicine physicians, also frequently confront malpractice claims related to diagnostic errors [5,6]. Internal medicine covers a broad spectrum of complaints and diseases, which can contribute to higher diagnostic uncertainty in patients presenting to internal medicine with undiagnosed conditions [3,7]. Therefore, more complex and difficult diagnostic decisions are required in internal medicine, which can result in higher susceptibility to diagnostic errors [3].

Diagnostic errors are usually related to multifactorial causes such as system-related errors; cognitive errors; and patient factors, including challenging disease presentations [8-10]. To date, while many studies have been conducted to address major system-related and cognitive errors using common measurements, patient factors, including challenging disease presentations, have not been investigated as much. However, among challenging disease presentations, atypical presentations have been increasingly recognized for their significant impact on diagnostic errors [11-15]. Atypical presentations are described as “a shortage of prototypical features that are most frequently encountered in patients with the disease, features encountered in advanced presentations of the disease or simply features of the disease commonly listed in medical textbooks” [16], which can make diagnosis more challenging and distract the diagnostic process. Indeed, atypical presentations were reported to be associated with a higher prevalence of diagnostic errors compared to typical presentations [12-14], and a pilot systematic review of case reports of diagnostic errors suggested that higher numbers of contributing factors were detected in the cases with atypical presentations compared to the cases without atypical presentations [15]. Moreover, a previous study showed that the prevalence of diagnostic errors in patients with atypical presentations of stroke was smaller when cared for by health care providers who are familiar with atypical presentations of stroke [17]. Although the results cannot be easily generalized to other conditions, developing strategies to prevent progression from an atypical presentation to diagnostic error seems to be a promising approach to improving patient safety [11,12].

Atypical presentations seem to be especially an important issue for diagnostic errors in internal medicine: first, atypical presentations may be commonly observed (up to approximately 30%) in the internal medicine department [13]; second, internal medicine physicians consider atypical presentations to be the most important contributor to diagnostic errors in their clinical practice [18]; and third, atypical presentations can be an important contributing factor to a higher prevalence of diagnostic errors in internal medicine [10,13]. Therefore, a comprehensive understanding of the association between atypical presentations and diagnostic errors is required. Knowledge gaps in this area persist possibly due to the lack of consensus on the definition and measurement of atypical presentations and because a comprehensive conceptual model to understand how atypical presentations progress to diagnostic errors is still lacking.

A scoping review is more appropriate than a systematic review when the review aims to identify knowledge gaps, scope a body of literature, and clarify concepts. A scoping review is also more appropriate than a narrative review when clarification around a concept or theory is required [19]. From this perspective, a scoping review is more ideally suited to determine the body of literature on atypical presentation and diagnostic errors and identify any gaps in knowledge [20]. Moreover, without a universal definition for atypical presentations, future studies about diagnostic errors may have systematic biases by excluding patients with atypical presentations at high risk for missed diagnostic opportunities [21]. Therefore, this scoping review will also facilitate future studies by developing practical criteria and measurements for atypical presentations.

Throughout this scoping review, the key terms such as diagnostic errors, atypical presentations, and internal medicine are defined as follows:

- Diagnostic errors—defined as the failure to (1) establish an accurate and timely explanation of the patient’s health problems or (2) communicate that explanation to the patient [1], which also include delayed, wrong, and missed diagnosis [10].
- Atypical presentations—defined as patient demographics (eg, age, sex, and race); symptoms and signs; test results; or clinical course, including the response to treatment, deviated from the prototypical patterns for the final diagnosis.
- Internal medicine—defined as a medical specialty concerned with the diagnosis and treatment of diseases of the internal organ systems of adults.

To develop a new definition and useful criteria to identify atypical presentations that are at high risk of diagnostic errors, this scoping review aims to identify and present the available information regarding the definitions and measurements for atypical presentations in the evidence sources about diagnostic errors in internal medicine.

The primary review question is “What definitions and measurements have been used to identify atypical presentations in the studies investigating diagnostic errors in adult patients in internal medicine?” The subquestions are “What specific diseases have been targeted by the studies investigating the association between atypical presentations and diagnostic errors in adult patients in internal medicine?” and “What specific types of atypical presentations have been reported as relevant to diagnostic errors in adult patients in internal medicine?”

## Methods

### Study Design

The proposed scoping review will be conducted in accordance with the Joanna Briggs Institute methodology for scoping reviews [22,23] and in line with the PRISMA-ScR (Preferred Reporting Items for Systematic Reviews and Meta-Analyses Extension for Scoping Reviews) [24].

### Inclusion Criteria

#### Participants

This review will consider studies that include adult patients. We will exclude studies that focus on children.

#### Concept

This review will consider studies that explore the association between atypical presentations and diagnostic errors (using any clear definition or criteria or measurement to identify atypical presentations and diagnostic errors). We will exclude studies that investigate only atypical presentations or diagnostic errors.

#### Context

This review will consider studies that focus on the setting of internal medicine.

#### Types of Sources

This scoping review will consider quantitative, qualitative, and mixed methods study designs for inclusion. In addition, systematic reviews and text and opinion papers will be considered for inclusion in the proposed scoping review. Case reports, case series, and conference abstracts will be excluded.

### Search Strategy

The search strategy will aim to locate both published and unpublished primary studies, reviews, and text and opinion papers. An initial limited search of MEDLINE (PubMed) and Science Citation Index Expanded (Web of Science) was undertaken to identify papers on the topic. The text words contained in the titles and abstracts of relevant papers, and the index terms used to describe the papers, were used to develop a full search strategy for MEDLINE (PubMed; Multimedia Appendix 1). The search strategy, including all identified keywords and index terms, will be adapted for each included information source. The reference lists of papers included in the review will be screened for additional papers. Papers published in all languages will be included. Papers indexed from database inception to December 31, 2023, will be included. The databases to be searched include MEDLINE (PubMed), Science Citation Index Expanded (Web of Science), CINAHL, Embase, Google Scholar, and Cochrane Library. Sources of unpublished studies and gray literature to be searched include medRxiv*.*

### Study or Source of Evidence Selection

Following the search, all identified records will be collated and uploaded into Covidence (Covidence), and duplicates will be removed. Following a pilot test, titles and abstracts will then be screened by 2 independent reviewers (YH and RK) for assessment against the inclusion criteria for the review. Potentially relevant papers will be retrieved in full, and their citation details imported into Covidence. The full text of selected citations will be assessed in detail against the inclusion criteria by 2 independent reviewers (YH and RK). Reasons for exclusion of full-text papers that do not meet the inclusion criteria will be recorded and reported in the scoping review. Any disagreements that arise between the reviewers at each stage of the selection process will be resolved through discussion or with a third reviewer (MY). Interrater reliability will be monitored and reported. The results of the search will be reported in full in the final scoping review and presented in a PRISMA (Preferred Reporting Items for Systematic Reviews and Meta-Analyses) flow diagram [25].

### Data Extraction

Data will be extracted from papers included in the scoping review by 2 independent reviewers (YH and RK) using a data extraction tool developed by the reviewers. The data extracted will include specific details about the patient characteristics (eg, age, sex, and disease), the definitions and measuring methods for atypical presentations and diagnostic errors, clinical settings (eg, department and outpatient or inpatient), type of evidence source, and the association between atypical presentations and diagnostic errors relevant to the review question. A draft extraction tool is provided in Table 1. The draft data extraction tool will be modified and revised as necessary during the process of extracting data from each included paper. Modifications will be detailed in the full scoping review. Any disagreements that arise between the reviewers will be resolved through discussion or with a third reviewer (MY). Authors of papers will be contacted to request missing or additional data, where required.

**Table 1:** Data extraction instrument for the scoping review. The scoping review will include published or unpublished studies indexed in databases until December 2023; the scoping review will include quantitative, qualitative, and mixed methods study designs; systematic reviews; and opinion papers. The target population is adult patients cared for in internal medicine settings without restriction on disease, location, or time frame.

			Data extraction
**Evidence source information**			
	Author	✓	
	Year	✓	
	Country	✓	
	Aim	✓	
	Study type or source	✓	
**Population**			
	Participants	✓	
	Age (years)	✓	
	Sex	✓	
	Target disease	✓	
**Context**			
	Setting	✓	
**Concept**			
	Details of the definitions and measurements to identify atypical presentations	✓	
	Details of the definitions and measurements to identify diagnostic errors	✓	
	Description or statistical results about the association between atypical presentations and diagnostic errors	✓	
	Details of the characteristics related to atypical presentations	✓	

### Data Analysis and Presentation

The extracted data will be presented in tabular format with descriptive statistics, and from this, the key components or types of atypical presentations—the purpose of this scoping review—will be identified to develop new criteria to identify atypical presentations for future studies of diagnostic errors. Developing the new criteria will follow the Joanna Briggs Institute guidance for a basic qualitative content analysis approach (following an inductive approach) [26]. In addition, we will classify the sources of evidence into 2 categories: disease-specific or generic studies to identify diseases and settings where the current evidence is lacking. Finally, we will list the specific types of atypical presentations highly associated with diagnostic errors.

## Results

As of January 2024, a literature search through multiple databases is ongoing. We will complete this study by December 2024.

## Discussion

### Expected Findings

This scoping review aims to identify and present the available information regarding the definitions and measurements for atypical presentations to inform a new definition and criteria to identify atypical presentations at high risk of diagnostic errors. To maximize the quality of this scoping review, we will follow the updated scoping review guidelines, use multiple databases, and develop search strategies in consultation with experienced and skilled librarians. In addition, our review group includes expertise in diagnostic error research and has experience in developing new theories and concepts related to the diagnostic process. This scoping review aims to provide a rigorous evidence summary to describe how atypical presentations have been defined and measured in prior literature and use these findings to develop new criteria to identify atypical presentations at high risk for diagnostic errors. Findings will also inform the development of a comprehensive conceptual model to understand the associations between atypical presentations and diagnostic errors in internal medicine.

### Comparison With Prior Work

In a systematic review focused on diagnostic errors in primary care, Kostopoulou et al [16] defined atypical presentations as “a shortage of prototypical features that are most frequently encountered in patients with the disease, features encountered in advanced presentations of the disease or simply features of the disease commonly listed in medical textbooks”; however, this definition has not been applied in subsequent research investigating the association between atypical presentations and diagnostic errors due to several limitations. For instance, the difficulty in defining the gold standard classic textbook disease descriptions may be one of the possible reasons that the definition has not been used. Current studies use disease-specific criteria for atypical presentations to detect and measure atypical presentations at high risk of diagnostic errors [12]. However, such an approach also has limitations: understanding of certain atypical presentation features of a disease evolves over time, and these features might not stand the test of time. An example of this is the loss of taste, which was recognized as an atypical symptom of COVID-19 during the early stages of the pandemic; however, as we learn more about the disease, the loss of taste is no more considered atypical for COVID-19. This scoping review will clarify the gaps and complexity in each disease’s diagnostic criteria to diagnose atypical presentations and suggest a more refined approach that accounts for the limitations of prior definitions. The implications of our findings will be significant, indicating the potential for improving diagnostic processes and diagnostic accuracy by applying our new criteria.

### Limitations

This scoping review will have several limitations. First, this scoping review will not include studies outside of internal medicine, and findings may not generalize to specialties outside of internal medicine. Furthermore, the evolving nature of diagnostic criteria and practices in internal medicine necessitates ongoing revision and validation of our proposed definitions and criteria. Second, although we will search for unpublished studies and gray literature, we may overlook some, potentially bringing bias to our understanding and conclusions. Third, confirmation bias can distort the findings in this scoping review because each study related to diagnostic errors may have overlooked atypical presentations. However, the upcoming new criteria from this scoping review can be expected to increase sensitivity to detect atypical presentations and reduce confirmation bias in future studies. Fourth, defining “atypical presentation” itself may present challenges, as evidenced by the difficulty in clearly distinguishing between concepts such as atypical presentations, phenotype, and nonspecific symptoms, as well as the challenge in defining the degree of atypicality and the boundary between typical and atypical. However, this is precisely the reason this research needs to be done because these issues need further clarification. Definitions and distinctions of these concepts will become clearer as a result of this exploration.

### Conclusions

This scoping review can provide a rigorous evidence summary to describe how atypical presentations have thus far been defined and measured and help develop new criteria to identify atypical presentations at high risk for diagnostic errors in internal medicine. Developing the new criteria is expected to facilitate the development of a comprehensive conceptual model to understand the associations between atypical presentations and diagnostic errors in internal medicine. Additionally, such criteria can reduce the systematic selection biases in future diagnostic error studies that evaluate patients with atypical presentations. Review findings will highlight the need for further research to validate and refine these criteria, aiming to improve diagnostic processes and outcomes for patients with atypical presentations.

## Appendix

Multimedia Appendix 1 Search strategy.

## Abbreviations

PRISMA: Preferred Reporting Items for Systematic Reviews and Meta-Analyses
PRISMA-ScR: Preferred Reporting Items for Systematic Reviews and Meta-Analyses Extension for Scoping Reviews

## Data Availability

Data sharing is not applicable to this paper as no data sets were generated or analyzed during this study.
