# Supplementary material for: Definitions and Measurements for Atypical Presentations at Risk for Diagnostic Errors in Internal Medicine: Protocol for a Scoping Review
Source: JMIR Res Protoc. 2024 Mar 25;13:e56933. doi: 10.2196/56933 (PMC11002735; doi:10.2196/56933)
Supplement: Multimedia Appendix 1 [file resprot_v13i1e56933_app1.docx]

MEDLINE (PubMed)

Search conducted on January 10th, 2024*.*

| **Search** | **Query** | **Records retrieved** |
| --- | --- | --- |
| #1 | "Diagnostic Errors"[MeSH Terms] OR "delayed diagnosis"[MeSH Terms] OR "diagnostic error*"[Text Word] OR "misdiagnos*"[Text Word] OR "delayed diagnos*"[Text Word] OR "diagnostic delay*"[Text Word] OR "delay in diagnos*"[Text Word] OR "delays in diagnos*"[Text Word] OR "diagnostic safet*"[Text Word] OR "diagnostic failure*"[Text Word] OR "diagnostic mistake*"[Text Word] OR "inaccurate diagnos*"[Text Word] OR "diagnostic uncertainty"[Text Word] OR "diagnostic challenge*"[Text Word] OR "challenge-in-diagnosis"[Text Word] OR "challenges-in-diagnosis"[Text Word] OR "diagnostic pitfall*"[Text Word] OR "pitfall-in-diagnosis"[Text Word] OR "pitfalls-in-diagnosis"[Text Word] OR "diagnostic blind spot*"[Text Word] OR "diagnostic discrepanc*"[Text Word] OR "wrong diagnos*"[Text Word] OR "missed diagnos*"[Text Word] | 214,539 |
| #2 | ("cognitive error*"[Text Word] OR "cognitive failure*"[Text Word] OR "cognitive bias*"[Text Word] OR ("cognition"[MeSH Terms] AND "bias"[MeSH Terms]) OR ("anchoring"[Text Word] AND "bias*"[Text Word]) OR "ascertainment bias*"[Text Word] OR "availability bias*"[Text Word] OR "base-rate-neglect"[Text Word] OR "commission bias*"[Text Word] OR "confirmation bias*"[Text Word] OR "diagnostic momentum"[Text Word] OR "framing effect*"[Text Word] OR "fundamental attribution error*"[Text Word] OR "gambler's fallacy"[Text Word] OR "gamblers fallacy"[Text Word] OR "gender bias*"[Text Word] OR "hindsight bias*"[Text Word] OR "illusory correlation*"[Text Word] OR "status quo bias*"[Text Word] OR "wallpaper phenomenon*"[Text Word] OR "omission bias*"[Text Word] OR "order effect*"[Text Word] OR "outcome bias*"[Text Word] OR "overconfidence bias*"[Text Word] OR "value bias*"[Text Word] OR "playing the odd*"[Text Word] OR "posterior probability error*"[Text Word] OR "premature closure*"[Text Word] OR "sunk cost*"[Text Word] OR "unpacking principle*"[Text Word] OR "affective bias*"[Text Word] OR "zebra retreat*"[Text Word] OR "ambiguity effect*"[Text Word] OR "representativeness bias*"[Text Word] OR "unconscious bias*"[Text Word] OR "cognitive dispositions to respond*"[Text Word] OR "heuristic*"[Text Word] OR "heuristics"[MeSH Terms] OR "unconscious bias*"[Text Word] OR "missed opportunit*"[Text Word]) AND ("clinical decision making"[MeSH Terms] OR "clinical decision*"[Text Word] OR "clinical reasoning"[Text Word] OR "Decision Making"[Text Word] OR "Decision Making"[MeSH Terms] OR "diagnos*"[Text Word] OR "common disease*"[Text Word] OR "common symptom*"[Text Word] OR "typical disease*"[Text Word] OR "typical symptom*"[Text Word]) | 10,090 |
| #3 | #1 OR #2 | 223,576 |
| #4 | "atypical"[Text Word] OR "uncommon"[Text Word] OR "abnormal"[Text Word] OR "unusual"[Text Word] OR "nonclassical*"[Text Word] OR "non classical"[Text Word] OR "rare"[Text Word] | 1,616,171 |
| #5 | #3 AND #4 | 45,288 |
| #6 | "internal medicine"[Text Word] OR "general internal medicine"[Text Word] OR "general medicine"[Text Word] OR "hospital medicine"[Text Word] OR "internal medicine"[MeSH Terms] OR "hospital medicine"[MeSH Terms] OR "hospitalists"[MeSH Terms] OR "internist*"[Text Word] OR "hospitalist*"[Text Word] OR "physician*"[Text Word] OR "physicians"[MeSH Terms] OR "nurse practitioner*"[Text Word] OR "Nurse Practitioners"[MeSH Terms] |  |
| #7 | #5 AND #6 | 2,737 |
| #8 | 0001/01/01:2023/12/31[Date - Create] | 36,648,154 |
| #9 | #7 AND #8 | 2,734 |
| #10 | "case reports"[Publication Type] OR "letter"[Publication Type] OR "comment"[Publication Type] OR "case*"[Title] | 4,448,211 |
| #11 | #9 NOT #10 | 1,355 |
